# Supplementary material for: Content-rich biological network constructed by mining PubMed abstracts
Source: BMC Bioinformatics. 2004 Oct 8;5:147. doi: 10.1186/1471-2105-5-147 (PMC528731; doi:10.1186/1471-2105-5-147)
Supplement: Additional File 2 — The original results of the above study (non-essential files are deleted to keep the file size under the limit set by BMC bioinformatics). [file 1471-2105-5-147-S2.bz2 › chilibotAdditionalFile2/dip05/755ID8631671E18141/html/EXBB_EXBD.html]

 


 **EXBB** and **EXBD** 
  
Found 56 abstracts in PubMed, retrieved 05.  
 

 What does Google say? 
 PDF only 
| .edu only 

---

**Interactive relationship** (e.g. stimulation, inhibition, etc)

**Neutral relationship**- The aim of this review is to describe an in vivo assay of the interactions taking place in the Tol Pal or TonB  **ExbB**   **ExbD**  envelope complexes in the periplasm of Escherichia coli and between them and colicins or g3p protein of filamentous bacteriophages.  Ref: 12423784 Biochimie< MedlineTA>Biochimie,

**Non-interactive relationship** (e.g. studied together, co-existance, homology, etc.)

- Reverse transcription RT PCR, using RNA from organisms grown under iron replete and iron restricted conditions, revealed that tonB,  **exbB** ,  **exbD** , tbpB and tbpA are transcribed as a single unit with expression being up regulated in response to iron restriction.  Ref: 12742718 Vet Microbiol, 2003
- PCR approaches allowed the amplification, cloning and sequencing of A. suis tonB,  **exbB** ,  **exbD** , tbpB and tbpA homologues.  Ref: 12742718 Vet Microbiol, 2003
- In gram negative organisms, high affinity transport of iron substrates requires energy transduction to specific outer membrane receptors by the TonB  **ExbB**   **ExbD**  complex.  Ref: 12562789 J Bacteriol, 2003
- The Ton system consisting of the proteins TonB,  **ExbB**  and  **ExbD**  is required for colicin and microcin import.  Ref: 12423780 Biochimie< MedlineTA>Biochimie, 2003
- The energy is transduced to FecA by the inner membrane complex, TonB,  **ExbB** , and  **ExbD** .  Ref: 12618451 J Bacteriol, 2003
